# Importance of fish for food and nutrition security among First Nations in Canada

**DOI:** 10.17269/s41997-021-00481-z

**Published:** 2021-06-28

**Authors:** Lesya Marushka, Malek Batal, Constantine Tikhonov, Tonio Sadik, Harold Schwartz, Amy Ing, Karen Fediuk, Hing Man Chan

**Affiliations:** 1 First Nations and Inuit Health Branch, Indigenous Services Canada, Ottawa, Canada; 2 Département de nutrition, Faculté de médecine, Université de Montréal, Pavillon Liliane de Stewart, C.P. 6128, succ. Centre-Ville, Montréal, QC H3T 1A8 Canada; 3 Centre de recherche en santé publique de l’Université de Montréal et du CIUSS du Centre-sud-de-l’Île-de-Montréal (CReSP), 7101 Avenue du Parc, Montréal, QC H3N 1X7 Canada; 4 Assembly of First Nations, 55 Metcalfe Street, Suite 1600, Ottawa, ON K1P 6L5 Canada; 5 First Nations Food, Nutrition and Environment Study, University of Ottawa, 30 Marie Curie, Ottawa, ON K1N 6N5 Canada; 6 Department of Biology, University of Ottawa, 30 Marie Curie, Ottawa, ON K1N 6N5 Canada

**Keywords:** First Nations, Food security, Traditional food, Fish consumption, Nutrient intake, Gender difference, Premières Nations, sécurité alimentaire, aliments traditionnels, consommation de poisson, apport en nutriments, différence entre les sexes

## Abstract

**Objective:**

To investigate the relationships between fish/seafood consumption patterns and food security status among First Nations (FN) communities in Canada. We estimated the contribution of fish/seafood to daily nutrient requirements. Barriers to traditional food (TF) access including fish were summarized.

**Methods:**

Data were collected by the First Nations Food, Nutrition and Environment Study (2008–2018). The sample of this participatory study comprised 6258 randomly selected FN adults. Fish/seafood consumption was estimated with a food frequency questionnaire. Food security status was assessed with the Household Food Security Survey Module. The contribution of fish/seafood to protein, n-3 fatty acid, vitamin (A, B12, D, niacin) and mineral (selenium, zinc) requirements was assessed by comparison to Dietary Reference Intakes.

**Results:**

Regional differences were observed in fish/seafood consumption patterns and their relationship with food security status. In the eastern regions (Ontario, Quebec/Labrador and the Atlantic region), consumption of fish/seafood and other TF was significantly higher among food insecure compared with food secure FN participants. Severely food insecure men (particularly in British Columbia, Alberta, Quebec/Labrador and the Atlantic region) tended to eat a higher amount of TF, including fish/seafood, compared with food secure and moderately food insecure men, while no difference was observed in women. Fish/seafood provided good sources of selected nutrients. However, the high cost of harvesting equipment, industry-related activities and climate change reduce access to fish/seafood and other wildlife.

**Conclusion:**

Fish/seafood continues to be vital to the diet of FN communities. Focusing on policies that support FN increased access to fish/seafood has the potential to decrease food insecurity and support sustainable livelihoods. Future policies should focus on socio-economic determinants of food insecurity and support traditional harvesting and sustainable fisheries among FN communities.

**Supplementary Information:**

The online version contains supplementary material available at 10.17269/s41997-021-00481-z.

## Résumé

**Objectif:**

Étudier les relations entre les modèles de consommation de poissons/fruits de mer et l’état de la sécurité alimentaire chez les Premières Nations (PN) au Canada. Nous avons estimé la contribution des poissons/fruits de mer aux besoins quotidiens en nutriments. Les obstacles à l’accès aux aliments traditionnels (AT), y compris le poisson, ont été résumés.

**Méthodes:**

Les données ont été recueillies dans le cadre de l’Étude sur l’alimentation, la nutrition et l’environnement chez les Premières Nations (2008–2018). L’échantillon de cette étude participative comprenait 6 258 adultes PN sélectionnés au hasard. La consommation de poissons/fruits de mer a été estimée à l’aide d’un questionnaire de fréquence alimentaire. Le niveau de sécurité alimentaire a été évalué à l’aide du Module d’enquête sur la sécurité alimentaire des ménages. La contribution des poissons/fruits de mer aux besoins en protéines, en acides gras n-3, en vitamines (A, B12, D, niacine) et en minéraux (sélénium, zinc) a été évaluée par rapport aux apports nutritionnels de référence.

**Résultats:**

Des différences régionales ont été observées dans les modes de consommation de poissons/fruits de mer et leur relation avec le niveau de sécurité alimentaire. Dans les régions de l’Est (Ontario, Québec/Labrador et région atlantique), la consommation de poissons/fruits de mer et d’autres AT était significativement plus élevée chez les personnes vivant dans les ménages en situation d’insécurité alimentaire que chez celles en sécurité alimentaire. Les hommes en situation d’insécurité alimentaire sévère (en particulier en Colombie-Britannique, en Alberta, au Québec/Labrador et en région atlantique) avaient tendance à manger une plus grande quantité d’AT, y compris du poisson/fruits de mer, par rapport aux hommes en sécurité alimentaire et en insécurité alimentaire modérée, alors qu’aucune différence n’a été observée chez les femmes. Les poissons/fruits de mer ont fourni de bonnes sources de nutriments sélectionnés. Cependant, le coût élevé du matériel de chasse, les activités liées à l’industrie et le changement climatique réduisent l’accès aux poissons/fruits de mer et à d’autres espèces sauvages.

**Conclusion:**

Les poissons/fruits de mer continuent d’être essentiels à l’alimentation des PN. L’amélioration de l’accès aux poissons/fruits de mer a le potentiel de promouvoir la sécurité alimentaire et des moyens de subsistance durables. Les politiques futures devraient se concentrer sur les déterminants socio-économiques de l’insécurité alimentaire et soutenir la récolte traditionnelle et la pêche durable chez les PN**.**

## Introduction

For thousands of years, Indigenous Peoples (First Nations (FN), Métis, and Inuit) of Canada have relied on their deep understanding of the local environment and adaptive measures to live successfully off the land (Kuhnlein et al. 2013a, b). Traditional food systems are diverse across geographical regions and include a great variety of fish species, game meat, and plants. Consumption of traditional food remains fundamental to Indigenous Peoples’ cultural identity and spiritual well-being (Kuhnlein et al. 2013a, b). While traditional diets provide excellent sources of energy, protein, and micronutrients (Sheehy et al. 2015; Blanchet et al. 2020), activities involved in the acquisition, preparation and consumption of traditional food maintain a spiritual connection with nature, facilitate knowledge transfer and contribute to physical health and overall well-being of Indigenous Peoples (Egeland et al. 2001; Kuhnlein et al. 2013a, b).

Indigenous people in Canada are diverse by culture, histories, and homelands, with more than 70 Indigenous languages being spoken across Canada (Statistics Canada 2017b). FN represent the greatest share of the Indigenous people in Canada (58.4%) and include individuals who are members of a First Nation/Indian Band and those who are not, as well as those with and without registered or treaty Indian status under the Indian Act (Statistics Canada 2017a). Within the FN population, 744,855 (76.2%) have a registered or treaty Indian status, with 44.2% living on-reserve or in communities on Crown land. The Assembly of First Nations (AFN) recognizes 634 First Nations/Indian Bands, spread across provinces and territories in Canada (Statistics Canada 2017a).

Fish, which is consumed by the majority of FN in Canada (Chan et al. 2019; Batal et al. 2021a), has always been an essential part of their traditional diets. Fishing activities, such as hand-gathering, spearing, netting and angling, have been widely used in ceremonies and festivals and contribute to the physical fitness and overall well-being of FN (Long 2014). Beyond cultural benefits, fish/seafood contribute to nutrition security (i.e., attaining acceptable levels of recommended nutrients) (Pangaribowo et al. 2013) by supplying rich sources of high-quality protein, omega-3 polyunsaturated fatty acids (n-3 PUFA), essential vitamins and minerals (Jamieson et al. 2013; Marushka et al. 2019).

Over the past several decades, FN have been experiencing a nutrition transition when traditional nutrient-dense diets have been gradually replaced by store-bought food, high in calories, fats and sugar (Kuhnlein et al. 2004; Johnson-Down and Egeland 2012; Robidoux et al. 2012; Blanchet et al. 2020). This nutrition transition has been concomitant with lifestyle changes and increased rates of obesity, diabetes and cardiovascular disease (Batal and Decelles 2019; Riediger et al. 2014). Changes in social and food practices have been linked to the long-term impacts of colonization, which continues to negatively impact FN people’s lives due to ongoing socio-economic inequities, food insecurity, and limited access to traditional food and healthy store-bought food (Willows et al. 2008). Furthermore, rapid environmental changes, including climate change, urbanization, environmental contamination and degradation, affect the access to and availability of local traditional food species, which exacerbates the nutrition transition and food insecurity among FN (Ford et al. 2010).

As defined by the World Food Summit in 1996, food security exists “when all people, at all times, have physical and economic access to sufficient, safe and nutritious food that meets their dietary needs and food preferences for an active and healthy life” (FAO 1996). Food security among FN is characterized by access to both traditional and store-bought foods (Power 2008). The four dimensions of traditional food security were defined as follows: food access (i.e., access to traditional food), food availability and supply (i.e., the impacts of environmental exposure and climate change on safety, availability and supply of traditional food), and food utilization (i.e., knowledge about nutritional benefits of traditional foods) (Power 2008). Food insecurity (i.e., the inability to afford nutritionally adequate and safe foods) is highly prevalent among FN in Canada and is recognized as a severe public health issue (FNIGC 2012). While over half (54.2%) of on-reserve FN households in Canada experience food insecurity, 14.1% reported having to cut the size of their meals or skip meals due to a lack of monetary resources and are therefore in the category of severely food insecure (FNIGC 2012). In contrast, only 12.3% of Canadian households experienced some level of food insecurity, with 5.6% being categorized as moderately food insecure and 2.5% as severely food insecure (Tarasuk et al. 2013). Among FN, food insecurity is associated with compromised diet quality, poor general and mental health, and a weak sense of community belonging (Egeland et al. 2011).

Given that generally, fish is perceived to play an integral role in the overall well-being of FN, the goal of this study was to explore the relative importance of fish consumption in the context of food and nutrition security in FN across Canada. Specifically, we investigated the relationships between fish/seafood consumption patterns and food security status. We estimated the contribution of fish/seafood to the nutrient requirements. Barriers to the access and availability of traditional food, including fish/seafood, were also summarized.

## Methods

### Study population

Data used in this study were collected by the First Nations Food, Nutrition and Environment Study (FNFNES). The FNFNES was a 10-year participatory study (2008–2018) designed to collect regionally representative data on diet quality, food-related exposure to environmental contaminants, food security and health status of FN people living on reserves south of the 60^th^ parallel across Canada. The FNFNES was implemented in the eight AFN regions, including British Columbia (BC), Alberta (AB), Saskatchewan (SK), Manitoba (MB), Ontario (ON), Quebec (QC) and the Atlantic region (AT) (Chan et al. 2019). The sampling of FN communities was random and based on a combined ecozone/cultural area framework, which allowed the representation of diet diversity. The sampling proceeded in three stages: first, communities were randomly sampled within each of the eight AFN regions; second, 100 households were randomly sampled within each selected community; and third, one adult who was self-identified as being a FN person living on-reserve and aged 19 and older was asked to participate in the study. Sample weights were calculated to obtain representative estimates of the total population. The design weights were adjusted based on the assumption that the responding communities represent both responding and non-responding communities. Data were also adjusted for changes in population from 2008 to 2017. More detailed information on the participatory process and the weighting approach is published elsewhere (Chan et al. 2021).

The current study included data collected from 92 FN communities. Overall, 56 (60%) participating communities were located more than 50 km away from a service centre, while 17 (18%) had no year-round road access (fly-in/winter roads only). Based on the remoteness index (Alasia et al. 2017), FN communities were classified into four groups: zones 1–4 (Batal et al. 2021b). In total, 6487 participants aged 19 years and older were recruited to the study with an overall participation rate of 78%. Individuals who did not complete the Household Food Security Survey Module (HFSSM) were excluded from the analysis. The final sample was comprised of 6258 individuals. Further details on the study design and methodology can be found elsewhere (Chan et al. 2019, 2021).

### Ethics

This survey was conducted following the “Tri-Council Policy Statement: Ethical Conduct for Research Involving Humans” and, in particular, Chapter 9, regarding research involving the FN, Inuit and Métis Peoples of Canada. Ethical approval was granted by the Research Ethics Boards of Health Canada, the University of Northern British Columbia, the University of Ottawa, and the Université de Montréal. Informed consent was obtained from all participants (Chan et al. 2021). The FNFNES followed the First Nations principles of Ownership, Control, Access and Possession (OCAP®) (Chan et al. 2021).

### Data collection

Data were collected using in-person household interviews by trained community research assistants. All participating individuals completed a traditional food frequency questionnaire (FFQ), a dietary 24-h recall, a social/health/lifestyle questionnaire (SHL), and the HFSSM. The FFQ was used to collect data on locally harvested traditional food consumption during the four seasons in the past year. It included all identified traditional foods and was representative of each participating community (www.fnfnes.ca).

The SHL questionnaire collected information on age, sex, weight and height (reported and measured), physical activity level, smoking status, educational attainment, household size, employment status, self-perceived health status, source of income, traditional food gathering activities, access to traditional food, and factors preventing households from using more traditional food.

Barriers to traditional food use were examined with both open- and closed-ended questions. Specifically, with an open-ended question, participants were asked to describe their household’s main barriers to traditional food use. Answers were reviewed and grouped into several categories. For the closed-ended questions, participants were asked to select those major industries and types of infrastructure (mining, forestry, oil and gas, hydro, farming, roadways), or local commercial and non-commercial activities (outfitters, recreational harvesters), and regulations that limited traditional food harvesting activities. Participants were also asked to describe any significant climate change impacts over the last 10 years and on traditional food. The questionnaires can be found here: www.fnfnes.ca.

The adequacy of their traditional food supply over the last 12 months was examined with two proposed responses:

1. “We worried whether our traditional food would run out before we could get more.”
2. “The traditional food that we got just didn’t last, and we couldn’t get any more.”

Participants were provided with three options for answers: often, sometimes or never.

Food security information was collected with the income-related HFSSM adapted for Aboriginal households (Batal et al. 2021b). Based on responses to 18 questions (10 questions for adults’ status and an additional 8 questions for households with children), all households were classified into one of four categories: food secure, marginally, moderately and/or severely food insecure (Batal et al. 2021b). In this study, food secure and marginally food insecure groups were combined since these groups were comparable in terms of fish/seafood and other traditional food consumption patterns as well as socio-economic characteristics. Similarly, moderately food insecure and severely food insecure groups were combined for selected analyses when no significant differences were observed between the groups. It is important to note that the HFSSM reflects “household” food security status and not necessarily the status of a particular individual within the household, while the FFQ is a tool used to interview individuals.

### Fish and other traditional food consumption

Consumption of traditional food (grams/day) was estimated from the FFQ by totalling the number of days in the past four seasons when consumption of a particular food item was reported, then multiplying by the age- and gender-specific portion size of the corresponding traditional food item (estimated from the 24-h recall results) and divided by 360 days (four seasons of 90 days each). When portion size values could not be estimated due to the limited number of people reporting the use of a particular traditional food species on the 24-h recall, the average portion size was calculated from other FNFNES regions. If a traditional food was not reported to be consumed on the 24-h recalls, portion size values from the literature for these food species were used instead (Chan et al. 2019, 2021). Overall, 18% of respondents reported eating at least one traditional food on the day of the 24-h recall (Chan et al. 2019, 2021).

In this study, traditional foods were classified into five food subgroups: fish/seafood (e.g., fish species, shellfish, seaweed and sea mammal species), land animals (land mammal species), birds (wild bird species), berries (wild berries species) and plants (wild nuts, wild plants, cultivated plants, tree foods and mushrooms).

### Estimation of nutrient intakes from fish

Nutrient composition data for fish/seafood species reported by FN participants were obtained from the Canadian Nutrient File, a national food composition database (Health Canada 2015), taking into account the preparation method (i.e., baked, broiled, boiled, or raw). The Dietary Reference Intake (DRIs), such as the Recommended Dietary Allowance (RDA) and Adequate Intake (AI), were used to assess the contribution of fish to nutrient requirements of protein, n-3 PUFA, vitamins (A, B12, niacin and D), and minerals (zinc and selenium) (Health Canada 2013). The DRIs are a comprehensive set of nutrient values for healthy populations used for assessing and planning diets. The RDA is the average daily level of intake sufficient to meet the nutrient requirements of nearly all (97–98%) healthy people. AI is established when evidence is insufficient to develop an RDA and is set at a level assumed to ensure nutritional adequacy (Health Canada 2013; Otten et al. 2006).

Fish/seafood was considered as an “excellent source” of the nutrient if it contributed at least 20% or more of the recommended daily intake (DRI) of that particular nutrient, and a “good source” of the nutrient if it provided at least 10–19% of the recommended daily intake (IOM 2010).

### Statistical analysis

Descriptive statistics included the calculation of means with 95% confidence intervals (CI) for continuous variables and proportions (%) for categorical variables. Bivariate regression analyses were performed to examine differences in socio-economic variables by food security status. Multivariable regression was performed to assess whether fish/seafood and traditional food subgroup consumption differed by food security status. The regression models developed for regional analyses were adjusted for age, sex, physical activity, traditional harvesting activities, and remoteness index, while the model with all provinces combined was additionally adjusted for the regions. Consumers were defined as individuals reporting consuming more than 0 g/day of a respective traditional food item. The percentage contribution of fish/seafood to the recommendations of selected nutrients (DRIs) was calculated according to sex and age groups (Otten et al. 2006) by dividing the amount of a particular nutrient obtained from fish/seafood per day by its DRI (RDA or AI), then multiplying that number by 100. The body mass index (BMI) was calculated as weight (kilograms) divided by the square of height (metres). *p*-values less than 0.05 were considered statistically significant. Data analyses were performed with the statistical software package Stata, version 14.2 (StataCorp, College Station, TX, USA). All analyses used weightings to obtain representative estimates.

## Results

**Fig. 1:**
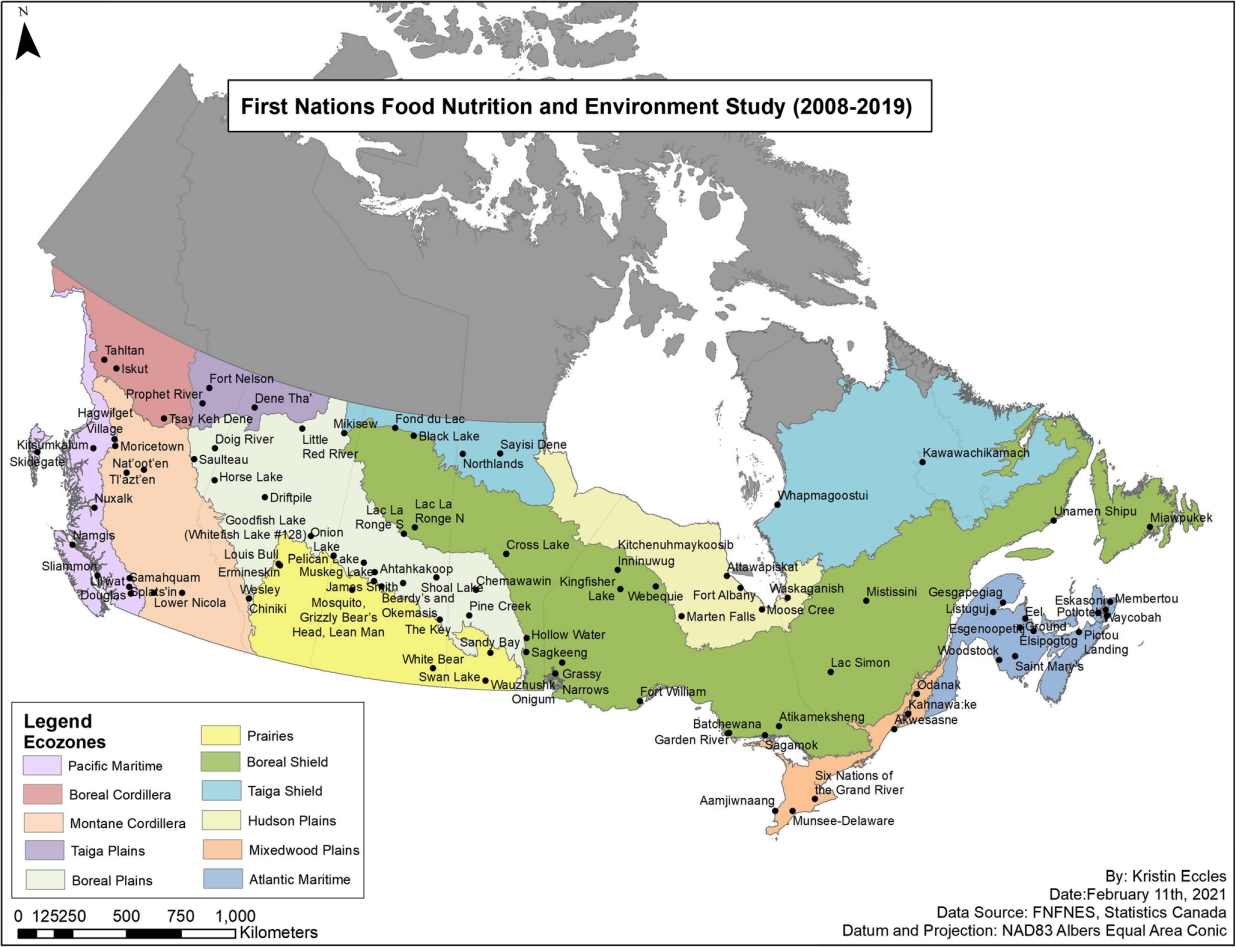
Map of participating communities, AFN regions and ecozones (Chan et al. 2021)

A total of 6258 individuals (2106 men and 4152 women) living in 92 FN communities located in 8 AFN regions across Canada participated in this study (Fig. 1).

Overall, almost half (47.9%) of participating households experienced some level of food insecurity. Among those, 10.4% were marginally food insecure, 28.8% were moderately food insecure, and 8.7% were severely food insecure households. Food insecurity was higher in western regions (BC (50.2%), AB (60.0%), SK (48.6%)) compared with the eastern regions (ON (38.8%), QC (48.5%) and AT (39.0%)). Overall, women tended to report higher food insecurity (51.1%) compared with men (40.9%). When remoteness index was considered, food insecurity was more prevalent in isolated communities (zone 4) (56%) and in communities located in zone 1 (49%) compared with the remote communities (zone 3) (43%) and those in zone 2 (45%) (Batal et al. 2021b).

Table 1 summarizes demographic and lifestyle characteristics by food security status. Overall, moderately and severely food insecure individuals were more likely to be younger than food secure individuals (43.2 years and 42.0 years vs 45.8 years, respectively), to be current smokers (56.1% and 63.0% vs 47.7%) and to report fair or poor health status more often (38.3% and 45.5% vs 30.2%). Also, a higher proportion of food insecure households had children under the age of 18 years (76.3% and 72.6% vs 64.0%) and lived in larger households (5.2 and 5.3 vs 4.6 people per household). Furthermore, food insecure individuals reported lower education attainment, a higher unemployment rate (34.1% and 38.0% vs 23.4%), and a higher reliance on social assistance (37.4% and 52.2% vs 19.6%) compared with food secure participants. Finally, whereas food secure participants tended to have at least one or more full-time workers in their households, food insecure participants did not.

**Table 1:** Socio-demographic characteristics of the FNFNES participants by food security status; First Nations Food, Nutrition and Environment Study (2008–2018)

	Food security status						*p* value
	Secure^a^		Moderately insecure		Severely insecure		
	Mean/*n*	95% CI/%	Mean/*n*	95% CI/%	Mean/*n*	95% CI/%	
*n* (%)	4061	62.0%	1632	28.8%	565	8.7%	
Age, mean (95% CI)	45.8	44.6–47.0	43.2	40.9–45.5	42.0	40.5–43.5	0.01
Females, *n* (%)	2654	60.0%	1118	31.0%	380	9.0%	0.05
Males, *n* (%)	1407	68.0%	514	24.1%	185	7.9%	
BMI, mean (95% CI)	30.8	30.3–31.3	31.3	30.6–32.0	29.4	28.1–30.8	0.09
Physical inactivity^b^, *n* (%)	2538	63.3%	1043	63.3%	360	60.9%	0.65
Current smokers, *n* (%)	1942	47.8%	1005	56.1%	391	63.0%	0.000
Health status, *n* (%)							0.000
Excellent/very good	1244	28.4%	364	23.6%	124	20.3%	
Good	1668	41.4%	677	38.2%	202	34.2%	
Poor/fair	1147	30.2%	591	38.3%	239	45.5%	
Household size, mean (95% CI)	4.6	4.3–4.9	5.2	5.0–5.4	5.3	4.8–5.7	0.001
Children under 18y, *n* (%)	2211	64.0%	1113	76.3%	332	72.6%	0.000
Education, *n* (%)							0.000
Less than high school	1420	38.7%	740	50.9%	240	52.3%	
High school	1311	34.0%	468	27.8%	185	30.6%	
Vocational training	325	10.0%	143	10.9%	61	11.2%	
Post-secondary education	741	17.3%	171	10.4%	55	5.9%	
Full-time workers, *n* (%)							0.000
0 FT	1619	31.8%	923	47.2%	398	56.2%	
1 FT	1419	33.4%	488	31.3%	125	29.9%	
2+ FT	1014	34.9%	215	21.5%	41	14.0%	
Unemployment, *n* (%)	1195	23.4%	683	34.1%	313	38.0%	0.000
Source of income, *n* (%)							0.000
Wages	2332	61.2%	649	41.5%	148	30.6%	
Social assistance	803	19.6%	663	37.4%	317	52.0%	
Pension	546	11.5%	158	10.0%	31	4.6%	
Workers compensation	256	4.7%	101	7.4%	40	7.7%	
Other	94	3.1%	41	3.7%	26	5.1%	

^a^Food secure group includes marginal food insecurity

^b^Physical inactivity includes self-reported sedentary and somewhat active lifestyle

*p* values correspond to bivariate regression analyses for continuous variables and Pearson’s chi-square tests for categorical variables

All estimates are weighted

The vast majority (95%) of all participating FN adults reported consuming at least one locally harvested traditional food in the prior year, while fish/seafood was consumed by about 71%. Based on calculations from the frequency of consumption, on average, FN participants (both consumers and non-consumers) ate about 44.6 g/day of traditional food, of which 15.4 g/day was fish/seafood. Consumption of traditional food was higher in remote (zone 3) (57.6 g/day) and fly-in only communities (zone 4) (67.3 g/day) compared with those with year-round road access within 50 km (zone 1) (40.0 g/day) and 50 to 350 km to the nearest service centres (zone 2) (43.9 g/day). The reliance on fish was greater in the fly-in only communities (23.6 g/day) and was comparable in zones 1, 2 and 3 (15.6 g/day, 13.3 g/day and 11.6 g/day, respectively). On average, men consumed approximately two times more fish/seafood compared with women (24.4 g/day vs 11.4 g/day, respectively).

**Fig. 2:**
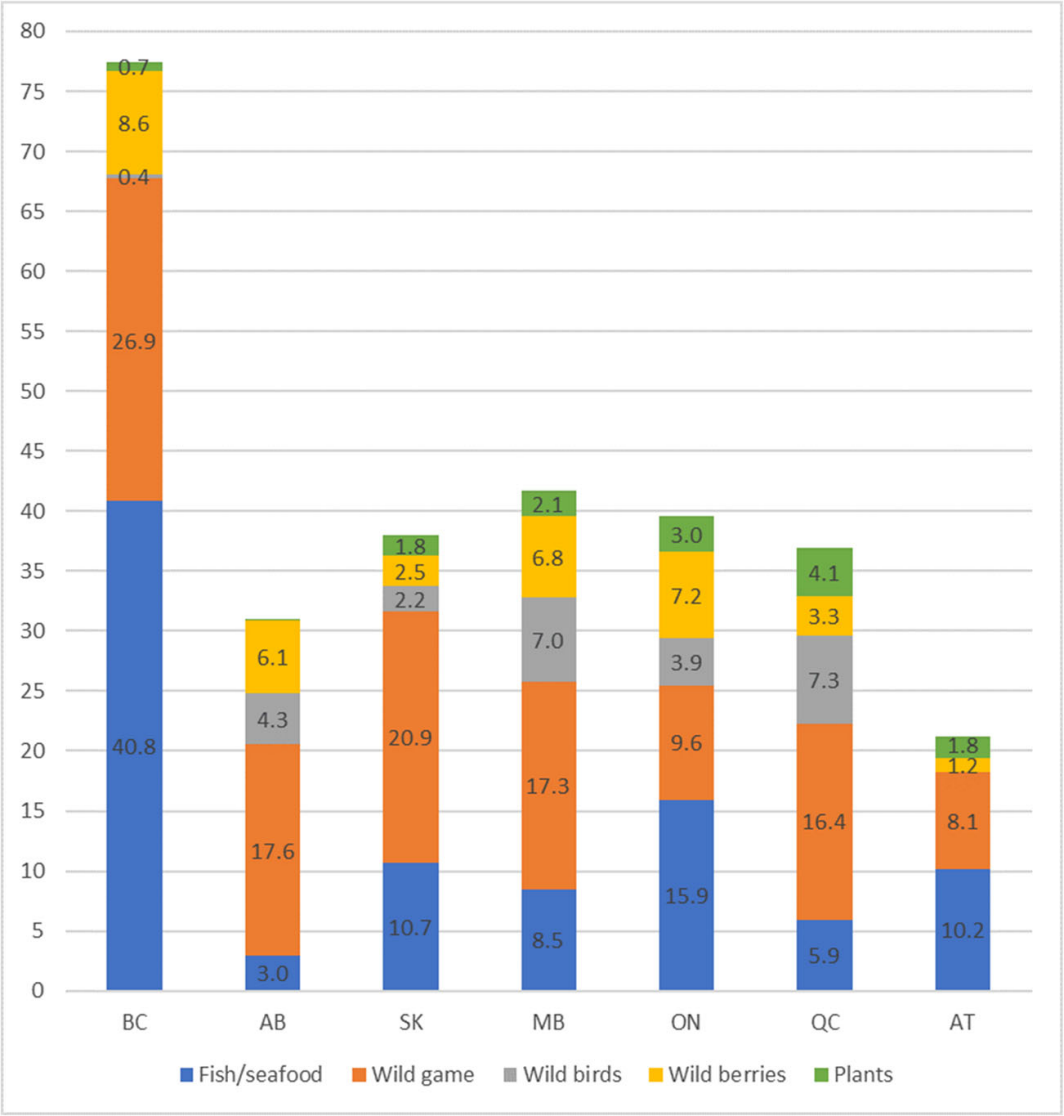
Mean consumption (g/day) of traditional foods (by subgroups) by region

Table 2 presents the mean intake of fish/seafood and other traditional food subgroups and the percentage of consumers in FN. The proportion of consumers of traditional food ranged from 85.1% (in AT) to 99.9% (in BC). Overall, the highest consumption of traditional food was reported by FN in BC (77.4 g/day), while the lowest traditional food intake (21.3g/day) was observed among FN in AT. The proportion of fish consumers ranged from 36.4% (in AB) to 94.0% (in BC). The mean intake of fish/seafood significantly varied across the regions. FN in BC reported the highest intake of fish/seafood (40.8 g/day) followed by ON (15.9 g/day), SK (10.7 g/day) and AT (10.2 g/day), while FN in AB consumed only 3 g/day of fish/seafood (Fig. 2). Overall, fish/seafood species represented 52.8% of the total traditional food intake in BC, 47.8% in AT and 40.1% in ON (Table 2). The lowest contribution of fish/seafood to total traditional food intake was in AB (9.5%) and QC (16%).

**Table 2:** Mean (population mean (consumers and non-consumers), based on the food frequency questionnaire and averaged across seasons, individuals aged ≥19 years) consumption (g/person/day) and percentage of consumers of traditional foods by region; FNFNES (2008–2018)

	British Columbia		Alberta		Saskatchewan		Manitoba		Ontario		Quebec		Atlantic	
	%^a^	Mean (95% CI)	%	Mean (95% CI)	%	Mean (95% CI)	%	Mean (95% CI)	%	Mean (95% CI)	%	Mean (95% CI)	%	Mean (95% CI)
**Fish/seafood**^b^	**94.0**	**40.8 (27.3–53.4)**	**36.4**	**3.0 (0.7–5.2)**	**51.7**	**10.7 (8.3–13.1)**	**85.0**	**8.5 (0.7–16.3)**	**72.2**	**15.9 (9.0–22.8)**	**77.0**	**5.9 (2.9–9.0)**	**67.4**	**10.2 (8.4–11.9)**
Land animals^c^	83.7	26.8 (12.1–41.7)	79.0	17.6 (6.8–28.4)	83.6	20.9 (13.1–28.7)	87.5	17.3 (7.5–27.1)	67.3	9.6 (5.4–13.8)	84.2	16.4 (11.0–21.8)	54.1	8.0 (4.2–11.8)
Birds	16.7	0.4 (0.2–0.7)	29.5	4.3 (1.4–7.1)	45.8	2.2 (1.7–2.6)	56.4	7.0 (0.7–14.7)	37.4	3.9 (1.6–6.2)	58.3	7.3 (1.0–15.6)	10.0	0.02 (0.01–0.03)
Berries	86.0	8.6 (6.0–11.1)	81.0	6.1 (2.7–9.2)	79.3	2.5 (1.5–3.4)	69.5	6.9 (4.1–9.1)	59.7	7.2 (4.4–10.0)	79.1	3.3 (1.9–4.6)	60.7	1.2 (0.8–1.6)
Plants^d^	37.6	0.7 (0.3–1.1)	41.9	0.05 (0.01–0.1)	51.6	1.8 (1.0–2.6)	30.3	2.1 (0.3–4.5)	56.3	2.9 (2.2–3.7)	51.4	4.1 (0.5–7.6)	45.7	1.8 (1.1–2.5)
Total TF	99.9	77.4 (56.7–98.2)	94.4	30.9 (15.4–46.5)	94.4	38.0 (30.9–45.1)	95.6	41.7 (18.6–64.9)	93.4	39.6 (25.3–53.8)	96.0	36.9 (21.7–52.2)	85.1	21.3 (15.6–27.0)
Contribution of fish/seafood to total TF		52.8%		9.5%		28.1%		20.4%		40.1%		16.0%		47.8%

^a^Percent of consumers

^b^Fish/seafood includes all locally harvested fish species and seafood (shellfish, seaweed and marine mammal species)

^c^Land animals includes all locally harvested land mammal species

^d^Plants include wild nuts, wild plants, tree foods, and mushrooms as well as cultivated plants

*TF*, traditional foods

Weighted estimates

**Fig. 3:**
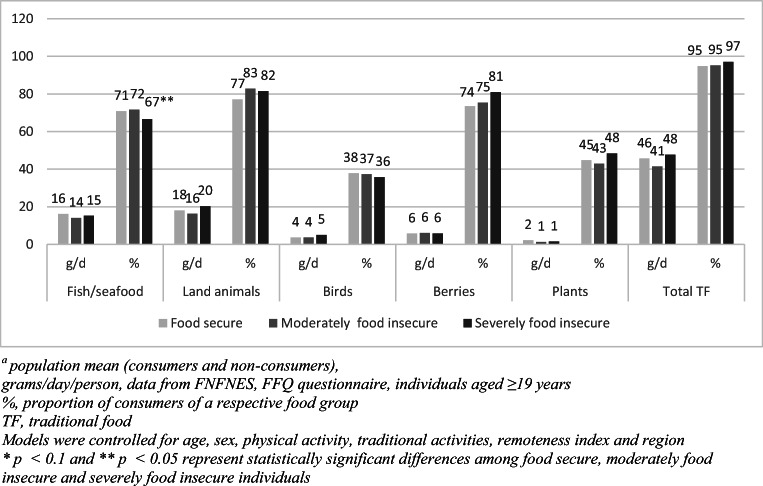
Mean^a^ intake and proportion of consumers of traditional foods by food security status in First Nations, all regions combined

We estimated mean intake (g/day) and the percentage of consumers (%) of fish/seafood and other traditional food subgroups by food security status. Figure 3 summarizes results for all regions combined. Estimates for each region are presented in Figures S1–S7 in the Supplementary Material. When data from all regions were combined, food secure and food insecure individuals did not significantly differ by the frequency and quantity of fish/seafood and other traditional food consumption. However, moderately food insecure individuals tended to consume a lower amount of fish/seafood and land animals, whereas severely food insecure participants consumed more fish/seafood and land animals compared with food secure FN participants. When analyses were stratified by gender, this tendency was observed in men but not in women. Overall, fish/seafood intake was 25.9 g/day, 19.5 g/day and 26.7 g/day among secure, moderately food insecure and severely food insecure men, respectively, while it ranged between 10.6 and 12.1 g/day by food security categories among women. At the regional level, a similar tendency appeared among FN adults in BC and AB. In contrast, in QC and AT, severely food insecure men reported higher consumption of fish/seafood compared with food secure men. Analyses stratified by the remoteness index showed that there were no significant differences in fish intake between food secure, moderately food insecure and severely food insecure groups in zones 1, 2 and 4. Specifically, fish consumption ranged from 12.1 to 17.9 g/day in zone 1, from 10.5 to 14.1 g/day in zone 2 and from 21.5 to 29 g/day in zone 4 across food security groups. In zone 3, however, fish intake was significantly higher among severely (20.6 g/day) and moderately (17.8 g/day) food insecure individuals compared with food secure participants (8.8 g/day).

At the regional level, traditional food consumption patterns notably differed among food secure, moderately food insecure and severely food insecure participants (Figures S1–S7). Among FN in BC, participants who experienced moderate food insecurity ate significantly lower amounts of fish/seafood (31 g/day), land animals (22 g/day) and total traditional food (62 g/day) compared with food secure participants (47 g/day, 29 g/day and 85 g/day, respectively). However, severely food insecure FN consumed, on average, more traditional food subgroups (e.g., 41 g/day, 34 g/day and 89 g/day, respectively) compared with both food secure and moderately food insecure respondents (Figure S1).

Among FN living in AB and SK (Figures S2-S3), fish/seafood consumption patterns did not significantly differ by food security status. However, in AB, the percentage of fish/seafood and bird consumers was lower among moderately and severely food insecure FN (40% vs 33% and 30% for fish/seafood; 34% vs 24% and 19% for birds) compared with the food secure group, while the proportion of individuals who consumed wild berries was higher in the severely food insecure group (93% vs 80%). In SK, a higher proportion of severely food insecure respondents relied on land animals (92% vs 81%) and plants (66% vs 49%) as compared with food secure individuals. Among FN in MB, the consumption of fish/seafood, land animals and birds was considerably lower among severely food insecure respondents (2 g/day, 13 g/day and 2 g/day, respectively) compared with moderately food insecure (10 g/day, 16 g/day and 6g/day) and food secure (8 g/day, 18 g/day and 8 g/day) FN adults (Figure S4). In contrast, among FN living in ON, QC and AT, both mean daily intake (g/day) and the percentage of consumers of the most traditional food subgroups increased with food insecurity status (Figures S5-S7).

**Fig. 4:**
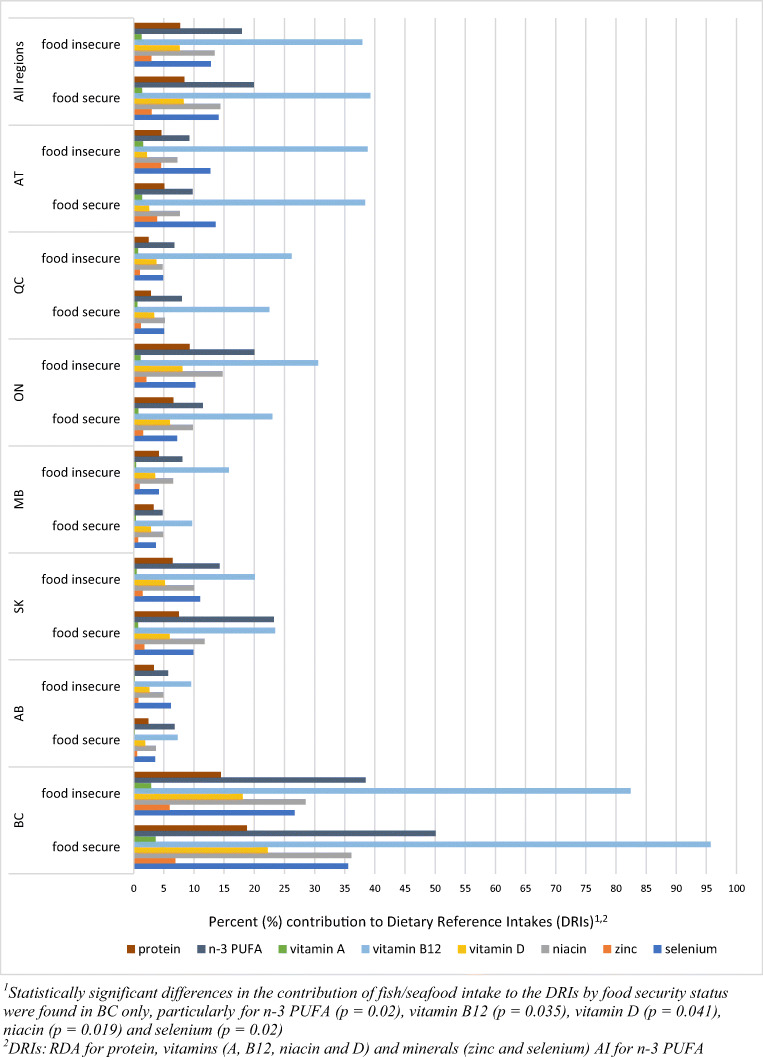
Contribution of fish and seafood to the recommended nutrient intake by food security status overall and by regions

Figure 4 presents results on the contribution of fish/seafood to the recommended daily intake of protein, n-3 PUFA, vitamins (A, B12, D and niacin) and minerals (zinc and selenium) by food security status. When all regions were combined, nutrient intake from fish/seafood did not significantly differ between food secure and food insecure participants. In both groups, fish/seafood supplied an excellent source of vitamin B12 (37.9% and 39.2%, respectively), and a good source of n-3 PUFA (17.9% and 19.9%), niacin (13.4% and 14.4%) and selenium (12.8% and 14.1%), and contributed up to 10% of protein, vitamin D, zinc and vitamin A. At the regional level, the highest contribution of fish/seafood to nutrient recommendations appeared in BC and was higher among food secure than food insecure individuals, particularly for n-3 PUFA, vitamin B12, vitamin D, niacin, and selenium (*p* values: 0.02, 0.035, 0.041, 0.019 and 0.022, respectively). The lowest contributions of fish/seafood to the requirements of selected nutrients were found in AB (<10% of the RDA or AI) due to relatively low fish/seafood consumption.

Table 3 summarizes responses on the availability of traditional foods and the engagement in traditional food harvesting activities in FN communities, which are also reported elsewhere (Batal et al. 2021a). Overall, a significantly higher proportion of food insecure compared with food secure individuals would like to have more traditional food in their diets; however, they experience a shortage in the traditional food supply. Analyses stratified by the remoteness index showed that FN respondents living in remote (zone 3) and isolated (zone 4) communities are more likely to report traditional food shortages (68.2% and 56.8%, respectively) compared with those FN individuals living in zones 1 and 2 (43–50%).

**Table 3:** Responses to questions related to accessibility and availability of traditional foods as well as traditional harvesting activity by food security status

	British Columbia (*n*=1065)			Alberta (*n*=594)			Saskatchewan (*n*=1008)			Manitoba (*n*=646)			Ontario (*n*=1376)			Quebec (*n*=556)			Atlantic (*n*=1013)			All regions (*n*=6258)		
	Total	Secure	Insecure	Total	Secure	Insecure	Total	Secure	Insecure	Total	Secure	Insecure	Total	Secure	Insecure	Total	Secure	Insecure	Total	Secure	Insecure	Total	Secure	Insecure
Households that want to have more TF	90.8	88.4	94.3*	77.7	68.4	88.1**	78.2	74.5	84.6**	68.5	63.1	77.4**	72.6	68.0	83.7**	83.7	83.5	84.7	60.3	54.4	72.8**	77.8	73.2	85.6**
Worried TF would run out before more could be obtained	67.7	61.7	76.4**	49.3	34.6	65.5**	42.2	33.2	57.4**	48.4	39.2	63.4**	28.3	21.1	45.8**	47.8	39.1	62.9**	25.7	18.0	42.8**	46.7	37.2	62.4**
Experienced TF shortages	72.0	65.1	82.1**	54.0	38.8	70.9**	47.9	38.4	63.7**	49.8	36.9	71.0**	31.0	21.4	53.3**	49.2	37.1	69.7**	29.7	22.1	46.3**	50.2	38.9	68.7**
Traditional activity by anyone in a household	74.9	77.5	71.0	64.8	63.6	66.2	62.1	60.0	65.9	58.9	56.5	62.8	70.2	68.3	74.8*	78.6	77.5	80.5	61.9	61.1	63.5	68.0	67.1	69.4
Traditional activity by a participant	59.9	63.2	55.0	51.7	53.0	50.4	46.7	43.5	52.2	43.9	43.6	44.3	56.0	54.6	59.2**	66.6	71.4	58.2**	47.8	47.1	49.3	53.8	54.4	52.7
Fishing activity by anyone in a household	58.1	63.6	50.3**	36.1	37.2	34.9	41.1	38.4	45.8*	47.7	45.3	51.6*	55.1	52.6	61.2**	59.7	58.2	62.4	48.7	48.0	50.3	50.0	50.0	50.0
Fishing activity by a participant	36.8	42.9	27.9*	23.9	29.0	18.2**	25.5	23.9	28.4	31.1	31.1	28.6	37.3	33.9	45.4**	35.6	39.5	28.7	31.7	31.2	32.7	31.9	33.7	29.1*

All values are percent, %

Traditional activity refers to fishing, hunting, setting snares for food, collecting wild plants or seafood, or planting a garden

*p < 0.1, **p < 0.05, *p* values represent statistically significant differences between secure and insecure groups within each region

Weighted estimates

Among traditional harvesting activities that were practiced by the majority (68%) of all FN households, fishing was the most common practice (50%). Fishing was more prevalent in BC (58%), QC (60%) and ON (55%). Participants living in remote (zone 3) and isolated (zone 4) communities engaged in fish-related harvesting activities more often (73.5% and 69%) than those living in zones 1 and 2; however, no differences were observed among food security groups. Food insecure households in SK, MB, ON and QC tended to engage in fishing more often than food secure households, while in BC and AB, fishing was less frequent among food insecure participants.

Table 4 summarizes barriers preventing FN households from using more traditionally harvested fish and other wildlife (overall and by food security status). At the individual level, three main barriers were identified: a lack of fisher/hunter, a lack of equipment/transportation, and a lack of time. The absence of a fisher/hunter in the household was reported by 18% of all respondents but was more prevalent among food insecure individuals in SK (29%), MB (34.1%) and QC (28.4%). In all regions, significantly higher proportions of food insecure households reported a lack of equipment and/or transportation as the main barrier to traditional food harvesting. Interestingly, lack of time, indicated by about 16% of respondents, was more frequently reported by food secure than food insecure participants across all regions.

**Table 4:** Barriers preventing households from using more traditional foods (overall and by food security status)

	British Columbia (*n*=1065)			Alberta (*n*=594)			Saskatchewan (*n*=1008)			Manitoba (*n*=646)			Ontario (*n*=1376)			Quebec (*n*=556)			Atlantic (*n*=1013)			All regions (*n*=6258)		
	Total	Secure	Insecure	Total	Secure	Insecure	Total	Secure	Insecure	Total	Secure	Insecure	Total	Secure	Insecure	Total	Secure	Insecure	Total	Secure	Insecure	Total	Secure	Insecure
Lack of a hunter in the household	8.0	7.5	8.6	18.7	14.7	23.3	29.0^§^	29.1	28.9	28.3^§^	24.7	34.1*	11.4	10.4	13.7	21.5^§^	17.6	28.4**	7.3	7.2	7.6	17.7	16	21.3**
Lack of equipment/transportation	33.7^§^	28.5	41.3*	25.4^§^	17.4	34.4**	15.7	11.2	23.5**	14.8	8.9	24.5**	14.1	7.3	30.6**	22.9^§^	18.9	30.0*	8.8	5.9	15.1**	20.9	14.7	30.9**
Lack of time	17.9	22.5	11.2*	12.8	13.7	11.9	15.0	16.1	13.2	10.5^§^	11.1	9.5	18.2	19.3	15.4	24.9	33.9	8.9*	10.6^§^	11.3	9.1	15.7	18.8	11.6
Government regulations	71.7^§^	68.0	77.2*	63.0^§^	61.7	64.3	45.8	42.6	51.5	67.0^§^	65.8	68.8	30.8	32.3	27.0	22.1	20.6	24.8	44.1	41.9	48.8*	51.4	48.7	56.0*
Forestry operations	67.1^§^	62.5	73.8*	63.2^§^	65.9	60.1	34.1	31.1	39.4	48.0	43.4	54.7*	31.1	30.3	33.0	46.6	41.4	55.5*	32.3	30.1	36.9	49.1	45.1	54.7**
Hydro operations	38.4	35.2	43.5*	39.5	37.4	41.8	18.6	18.1	19.4	55.8^§^	52.9	60.0	23.9	23.3	25.6	25.4	26.7	23.8	20.6	20.0	22.0	33.5	30.9	36.7**
Oil, gas and mining	42.0	43.4	39.8	68.2^§^	72.7	63.1*	36.4	33.5	41.9	27.0	24.0	31.4	27.6	23.7	36.7**	28.6	25.8	33.8	23.6	23.2	24.5	38.3	36.2	41.8
Climate change ^a^	66.0^§^	61.1	73.2**	46.1	41.4	51.2	38.1	35.9	42.0	48.9	43.5	57.7*	55.4	52.7	61.9*	51.1	52.8	48.1	42.4	39.7	48.3**	50.6	48.4	56.8**

^a^Climate change was perceived to decrease the availability of traditional food, increase the difficulty in getting traditional food, affect animals’ usual cycles or patterns and growth, and change fish run

Values are percent, %

Weighted estimates

*p < 0.1, ** *p* < 0.05, *p* values represent statistically significant differences between food secure and insecure groups within each region

^§^Significantly different from other regions

Other constraints that were reported to limit traditional harvesting activities included governmental regulations, natural resource industries (hydro/forestry operations, oil, gas and mining) and climate change, which were reported by more than half (51.4%) of all households (Table 4). More FN adults living in the western regions (BC, AB and MB) identified that governmental regulations and natural resource industries negatively impacted their engagement in harvesting activities (66.0–77.2%) compared with the eastern regions, such as ON, QC and AT (22.1–48.8%). When remoteness index was considered, government restrictions were mostly perceived by FN respondents living in zones 1 and 2 (54–58%) while forestry/hydro and mining operations were identified as a barrier more often by FN participants living in remote communities (zone 3) ranging from 56% to 78%.

Furthermore, over half (50.6%) of FN participants mentioned that they noticed climate change in their traditional territories, which was perceived to increase the difficulty in getting traditional food. Impacts of climate change on availability of and accessibility to traditional food, including fish/seafood, were perceived more frequently by food insecure FN adults living in BC (73.2%), ON (61.9%), MB (57.7%) and AT (48.3%) than by food secure FN individuals residing in these regions.

## Discussion

In response to the World Food Summit Plan of Action, Canada developed the Action Plan for Food Security to develop economic, social and environmental programs and policies and to promote national and international food security (Government of Canada 1998). Nevertheless, food insecurity continues to be a critical public health issue. More than one third of households (37.5%) among FN people living on reserves south of the 60^th^ parallel in Canada experience moderate or severe food insecurity, which is three to five times higher than in the general Canadian population (8.1%). These findings are similar to those from other studies reporting disproportionally higher rates of food insecurity among Indigenous people compared with the general Canadian population (Skinner et al. 2014; Tarasuk et al. 2013).

The health and well-being of FN are closely linked to foods and diets provided by local food systems. Fish and marine sources are particularly important since they are naturally rich in omega-3 fatty acids, high-quality protein, plus several minerals and vitamins, and thus, promote food and nutrition security for FN. In this study, we found significant regional differences in fish/seafood consumption patterns by food insecurity status. In the eastern regions (ON, QC and AT), consumption of fish/seafood was significantly higher among food insecure than food secure FN individuals, suggesting that individuals with limited availability and access to healthy store-bought foods tend to rely more on traditional foods, particularly fish, for their subsistence. In fact, a higher proportion of food insecure than food secure respondents in ON and QC reported engaging in traditional harvesting activities, including fishing. In contrast, FN respondents residing in the western regions (BC, AB and SK) consumed, on average, a similar amount of fish/seafood and other traditional food regardless of their food security status, while in MB, food insecure individuals consumed less fish/seafood than food secure people. Given a relatively higher prevalence of food insecurity and lower socio-economic status among FN in AB, SK and MB (Batal et al. 2021b), these results may indicate that financial constraints limit their access to both market and traditional foods. In remote communities (zone 3), food insecure participants consumed more fish compared with food secure respondents, whereas no differences were observed in communities with year-round road access (zones 1 and 2) and in isolated communities (zone 4). Similarly, fish-related activities did not differ among food security groups across zones. Further analyses are needed to examine factors contributing to the variations in fish consumption patterns and fishing practices among food secure and food insecure households.

Over one third of food insecure individuals living in the western regions did not have adequate access to equipment for fishing and hunting due to the high cost. The second significant barrier was the absence of a hunter/fisher in the family, reported by about 30% of participants, particularly in AB, SK and MB. Previous research showed that the high cost of harvesting equipment was among the primary factors preventing households from acquiring more traditional food (Nelson et al. 2005; Lambden et al. 2006; Goodman 2008; Kuhnlein et al. 2013a, b). Data from a study with Yukon FN, Dene/Métis and Inuit reported that up to 50% of respondents had inadequate access to fishing and hunting equipment, and up to 46% of participants said they could not afford to go hunting or fishing (Lambden et al. 2006). Among Yukon FN, Dene/Métis and Inuit living in Arctic Canada, only 40–45% of women’s households had access to harvesting equipment, with 11% and 29% of women reporting that hunting and fishing, respectively, were too expensive for their families (Goodman 2008). Also, participants from a study with the Dene Nation reported that the high costs of fuel and equipment have been limiting their ability to go out on the land to harvest traditional food (Kuhnlein et al. 2013a, b).

Our study also observed gender differences in traditional food consumption patterns by food security status. Severely food insecure men (particularly in BC, AB, QC and AT) tended to eat a higher amount of traditional food, including fish/seafood, compared with food secure and moderately food insecure men, while no difference was observed in women. This may suggest that food insecure men (but not women) are more likely to go hunting and fishing when they experience a lack of food (Marushka et al. 2018). This may also indicate that these female respondent households may be single-headed households with no fisher/hunter in their families. These findings need to be further investigated. Overall, more women than men reported food insecurity. This finding is consistent with a previous study and may indicate that male participants are more likely than female participants to under-report the level of food insecurity (Matheson and McIntyre 2013). Since women tend to take more responsibility in relation to foodstuff and preparing meals, they may have a better understanding of the food security issues of their families (Jung et al. 2017). Additionally, when there is a shortage of food in a household, females are more likely to be the first to cut or skip meals to ensure that other family members, particularly children, have access to sufficient food (Jung et al. 2017).

Our findings show that fish consumption provides important sources of essential nutrients. Nutrient intake from fish/seafood was higher among FN in BC, ON and SK, which reflects higher consumption of fish/seafood (by weight). This confirms the critical role of locally harvested fish in supporting nutritional health and food security in these FN communities. It should be noted, however, that 29% of FN respondents did not report eating any fish/seafood on the FFQ (including almost 64% of respondents from Alberta); thus, the contribution does not apply to all FN. Previous studies among Indigenous populations have also documented that traditional foods substantially contribute to micronutrient intakes (Sheehy et al. 2015; Blanchet et al. 2020).

Differences in traditional food consumption patterns reflect the diversity of traditional food systems across regions, cultural preferences, and the impacts of socio-economic and environmental factors. The vast majority of FN adults who participated in this study would like to have more traditional foods in their diets. However, high proportions of individuals who experience income-related food insecurity affirmed that they experience a shortage of traditional foods. This indicates that levels of accessibility and availability for traditional foods fall short of levels of demand by FN. Besides financial constraints that diminish the ability to obtain healthy market foods, FN experience challenges acquiring traditional foods. Our findings are consistent with the results from other studies, including a survey among Coast Salish people on Vancouver Island in British Columbia (Fediuk and Thom 2003). That study showed that levels of available traditional foods fall far short of levels desired by almost all respondents who wish to engage in traditional harvesting activities. The key barriers to greater use included government restrictions, environmental changes, poverty, privatization and traditional knowledge loss. In our study, barriers preventing FN respondents from the consumption of traditional foods differed across the regions. In particular, more FN living in the western regions reported governmental regulations, forestry/hydro operations, and oil, gas and mining as significant constraints to harvesting activities. Furthermore, FN across all regions reported concern about the impacts of climate change on their ability to use the land.

Limited access to fish and engagement in fish-related harvesting activities has significant implications for human health due to its crucial role in supplying essential micronutrients. Although some nutrients (i.e., protein, vitamin B12, niacin) can be obtained from alternative traditional foods and/or store-bought food, the intake of nutrients primarily derived from fish species (such as n-3 PUFA, vitamin D and selenium) is substantially diminished (Marushka et al. 2019). While n-3 PUFA are well known for their protective effects against cardiovascular disease (Mori 2014), vitamin D (Kulie et al. 2009) is essential for maintaining healthy bones and immune function, and selenium reduces the risk of cancer and autoimmune and thyroid diseases (Rayman 2000). Low intake of vitamin A, vitamin D, calcium, iron and magnesium among Canadian FN has been widely reported (Johnson-Down and Egeland 2012; Sheehy et al. 2015).

In Northern Canada, several hunter and harvester support programs have been developed to promote traditional harvesting activities. For example, the Nunavut Harvesters Support Program in Nunavut, the Inuit Hunting, Fishing and Trapping Support Program in Nunavik, and the Community Harvester Assistance Program and the Inuvialuit Harvesters Assistance Program in the Northwest Territories provide financial assistance to harvesters in the form of hunting equipment (snowmobiles, boats and all-terrain vehicles) and small supplies (fishing nets, camp stoves, sleeping bags, etc.) (NTI 2019). In James Bay, the Cree Hunters and Trappers Income Security Program provides an annual income to Cree FN, who regularly participate in harvesting activities (CHTISB 2019). In Manitoba, numerous initiatives, such as harvest support programs, traditional food education and nutrition school activities, land-based education programs and communities freezer programs, are implemented to support harvesters, to increase the access to traditional foods, to teach children and youth hunting skills and to incorporate traditional culture into healthy eating in northern FN communities (Food Matters Manitoba 2013). Recently, a new addition to the Nutrition North program, the Harvesters Support Grant, was launched (Government of Canada 2020). This grant supports the complete range of harvesting activities and traditions by reducing the high costs associated with traditional hunting and harvesting in northern Indigenous communities (Government of Canada 2020).

There are some limitations to this study. Intakes of nutrients from fish/seafood consumption were estimated based on the Canadian Nutrient File’s food composition data. Since the levels of some nutrients, such as n-3 PUFA and selenium, may vary within species and regions (Laird et al. 2018), the nutrient contents used in the study may contain intrinsic errors. The FNFNES data were collected over 10 years, which may have resulted in changes in traditional food consumption habits as well as in the effects of climate change on the local wildlife over these years. Finally, since fish/seafood consumption was estimated with the FFQ over the prior year, there is a potential for recall bias.

Food insecurity in FN communities presents a complex challenge and requires a multi-dimensional approach. Potential strategies, such as increasing access to traditional land and wildlife resources (i.e., protected rights to access lands and to harvest), traditional food subsidy programs directed to offset the high cost of hunting equipment, enhanced traditional knowledge transition from elders to younger community members, traditional food sharing with community members, and the transformation of fisheries management, would help to promote food sovereignty and sustainable livelihood in FN communities.

## Conclusion

Our findings show that on-reserve First Nations living south of the 60^th^ parallel in Canada experience very high rates of food insecurity. Traditional food systems, in particular, fish and seafood, remain essential to the contemporary diet of many FN. Fish consumption, for the majority of FN people, makes important contributions to nutritional health and food security. Several socio-economic and environmental barriers continue to prevent FN from fully taking advantage of traditional harvesting activities, including fishing. Improving access to fish and other wildlife has the potential to promote food security, sustainable livelihood and overall well-being among FN. Future policies and programs should focus on socio-economic determinants of food insecurity, support traditional harvesting activities and sustainable fisheries among FN communities, and preserve environments for improved access to traditional food.

## Supplementary information

ESM 1 (DOCX 50 kb)

## Data Availability

Data are owned by each participating community. The Assembly of First Nations is data custodian and any requests will be addressed to AFN through the corresponding author.

## Code availability

Analysis codes can be shared upon reasonable request.
