# Supplementary material for: Importance of fish for food and nutrition security among First Nations in Canada
Source: Can J Public Health. 2021 Jun 28;112(Suppl 1):64–80. doi: 10.17269/s41997-021-00481-z (PMC8239089; doi:10.17269/s41997-021-00481-z)
Supplement: Supplementary file 1 — (DOCX 50 kb) [file 41997_2021_481_MOESM1_ESM.docx]

Figure S1. Mean^a^ intake and proportion of consumers of traditional foods by food security status in British Columbia

*^a^ population mean (consumers and non-consumers),*

*g/d, grams/day/person, data from British Columbia FNFNES, FFQ questionnaire, individuals aged ≥19 years*

*%, proportion of consumers of a respective food group*

*TF, traditional food*

*Models were controlled for age, sex, physical activity, traditional activities and remoteness index*

** p value<0.1 and ** p value<0.05 represent statistically significant differences between food secure, moderately food insecure and severely food insecure individuals*

Figure S2. Mean^a^ intake and proportion of consumers of traditional foods by food security status in Alberta

*^a^ population mean (consumers and non-consumers),*

*g/d, grams/day/person, data from Alberta FNFNES, FFQ questionnaire, individuals aged ≥19 years*

*%, proportion of consumers of a respective food group*

*TF, traditional food*

*Models were controlled for age, sex physical activity, traditional activities and remoteness index,*

** p value<0.1 and ** p value<0.05 represent statistically significant differences between food secure, moderately food insecure and severely food insecure individuals*

Figure S3. Mean^a^ intake and proportion of consumers of traditional foods by food security status in Saskatchewan

*^a^ population mean (consumers and non-consumers),*

*g/d, grams/day/person, data from Saskatchewan FNFNES, FFQ questionnaire, individuals aged ≥19 years*

*%, proportion of consumers of a respective food group*

*TF, traditional food*

*Models were controlled for age, sex, physical activity, traditional activities and remoteness index*

** p value<0.1 and ** p value<0.05 represent statistically significant differences between food secure, moderately food insecure and severely food insecure individuals*

Figure S4. Mean^a^ intake and proportion of consumers of traditional foods by food security status in Manitoba

*^a^ population mean (consumers and non-consumers),*

*g/d, grams/day/person, data from Manitoba FNFNES, FFQ questionnaire, individuals aged ≥19 years*

*%, proportion of consumers of a respective food group*

*TF, traditional food*

*Models were controlled for age, sex, physical activity, traditional activities and remoteness index*

** p value<0.1 and ** p value<0.05 represent statistically significant differences between food secure, moderately food insecure and severely food insecure individuals*

Figure S5. Mean^a^ intake and proportion of consumers of traditional foods by food security status in Ontario

*^a^ population mean (consumers and non-consumers),*

*g/d, grams/day/person, data from Ontario FNFNES, FFQ questionnaire, individuals aged ≥19 years*

*%, proportion of consumers of a respective food group*

*TF, traditional food*

*Models were controlled for age, sex, physical activity, traditional activities and remoteness index*

** p value<0.1 and ** p value<0.05 represent statistically significant differences between food secure, moderately food insecure and severely food insecure individuals*

Figure S6. Mean^a^ intake and proportion of consumers of traditional foods by food security status in Quebec

*^a^ population mean (consumers and non-consumers),*

*g/d, grams/day/person, data from Quebec FNFNES, FFQ questionnaire, individuals aged ≥19 years*

*%, proportion of consumers of a respective food group*

*TF, traditional food*

*Models were controlled for age, sex, physical activity, traditional activities and remoteness index*

** p value<0.1 and ** p value<0.05 represent statistically significant differences between food secure, moderately food insecure and severely food insecure individuals*

Figure S7. Mean^a^ intake and proportion of consumers of traditional foods by food security status in Atlantic

*^a^ population mean (consumers and non-consumers),*

*g/d, grams/day/person, data from Atlantic FNFNES, FFQ questionnaire, individuals aged ≥19 years*

*%, proportion of consumers of a respective food group*

*TF, traditional food*

*Models were controlled for age, sex, physical activity, traditional activities and remoteness index*

** p value<0.1 and ** p value<0.05 represent statistically significant differences between food secure, moderately food insecure and severely food insecure individuals*
